# System Dynamics Modeling Can be Leveraged to Predict Critical Care Pathways and Costs for End Stage Renal Disease: US Population to 2020

**DOI:** 10.36469/9839

**Published:** 2015-07-22

**Authors:** Luca Fernandez, Christopher Koliba, Asim Zia, Katharine Cheung, Richard Solomon, Christopher Jones

**Affiliations:** 1 Community Development& Applied Economics University of Vermont, Burlington, VT; 2 Nephrology Unit University of Vermont College of Medicine; 3 Global Health Economics Unit, Center for Clinical and Translational Science University of Vermont College of Medicine

**Keywords:** epidemiology, modeling, transplantation, ckd, esrd, system dynamics

## Abstract

**Background:** End Stage Renal Disease (ESRD) accounts for 9% of Medicare spending, with the beneficiaries suffering from ESRD costing 7-9 times more than the average. This population is expected to continue to grow as a portion of Medicare beneficiaries. To provide clinicians and administrators with a greater understanding of the combined costs associated with the multiple critical care pathways for End Stage Renal Disease we have developed a model to predict ESRD populations through 2020.

**Methods:** A system dynamics model was designed to project the prevalence and total costs of ESRD treatment for the United States through 2020. Incidence, transplant and mortality rates were modeled for 35 age and primary diagnosis subgroups coursing through different ESRD critical care pathways. Using a web interface that allows users to alter certain combinations of parameters, several demonstration analysis were run to predict the impact of three policy interventions on the future of ESRD care

**Results:** The model was successfully calibrated against the output of United States Renal Data System’s (USRDS) prior predictions and tested by comparing the output to historical data. Our model predicts that the ESRD patient population will continue to rise, with total prevalence increasing to 829,000 by 2020. This would be a 30% increase from the reported 2010 prevalence.

**Conclusions:** Findings suggest that clinical care and policy changes can be leveraged to more effectively and efficiently manage the inevitable growth of ESRD patient populations. Patients can be shifted to more effective treatments, while planning integrating systems thinking can save Medicare’s ESRD program billions over the next decade.

## BACKGROUND

Providing care for patients with End Stage Renal Disease (ESRD) is one of the largest and least controlled healthcare costs in the United States.[1] Treatment of ESRD is also among the most complex in medicine, involving multiple common and intractable comorbidities and several intertwined treatment options. In the United States as a result of the ESRD Act of 1972, coverage for dialysis and transplantation is paid for under Medicare for all patients who require the treatment, regardless of age. What Congress intended as an emergency coverage measure for a relatively rare, but prohibitively expensive condition, quickly expanded under a more than tenfold increase in incidence and prevalence due to increases in the prevalence of kidney damaging comorbidities such as diabetes, as well as the for-profit dialysis industry.[1] In-center dialysis costs are $40,000-50,000 per year per patient. Additional costs related to managing comorbidities including anemia, diabetes, metabolic bone disease, hypertension and heart diseases, increases ESRD Medicare costs to $70-90,000 per year. This is in contrast to the average Medicare beneficiary costs of approximately $10,000, and the average cost of beneficiaries with six or more chronic conditions of $32,000.[2] While ESRD patients make up only 1% of Medicare beneficiaries, they account for almost 10% of expenditures. When elderly patients with chronic kidney disease are also included, the total Medicare costs approach 25% of total dollars spent.[3]

Medicare has made several attempts to reduce costs and manage quality: creating incentives for patients to choose self-dialysis or transplant in 1978; setting an adjusted per dialysis treatment payment rate in 1981, and bundling costs in 2003.[4] The Quality Incentive Program (QIP), which began in 2008, requires providers to meet performance measures for dialysis adequacy and anemia management, or Medicare will reduce their reimbursement by up to 2%.[5] Despite these measures, the cost continues to rise in response to the continued growth in ESRD incidence rate and prevalent population.

The last projection of ESRD patient population and costs was created in 2009 by the United States Renal Dara System (USRDS).[6] If effective policies for impacting the growth and cost of a complex condition such as ESRD are to be implemented, policy makers need not only up-to-date projections, but new tools for designing and simulating policy interventions. To meet this need, our team developed a System Dynamics (SD) model that allows for the simulation of interventions in the ESRD population. By changing the rates for several leverage point parameters such as incidence rate, the growth in incidence rate, the rate of kidney donations, and the use of peritoneal dialysis, a wide range of policy simulations can be run separately or simultaneously. SD modeling relies on simplifying complex environments by mapping the positive and negative feedback processes of patients between disease and cost states and a variety of other factors shaping critical care pathway decisions in the system. This method has been successfully implemented in the health care field, including modeling the impact of long term care capacity on elderly populations, a national level health care reform policy simulation, modeling pharmaceutical cost fluctuations for patients in Chinese hospitals, and many other health care applications dating back to the earliest development of SD modeling in the 1970's.[7,8,9,10]

Using this method we can answer several key research questions. The overarching question is: What will the population and rates of change of ESRD patients look like in 2020? Scenarios for several specific policy and critical care treatment pathways are posited, in which the following questions are asked: How does shifting the number of patients over 75 into conservative care pathways impact cost and mortality? How does doubling the kidney transplantation rate impact cost and mortality? How does shifting a significant percentage of patients from hemodialysis to peritoneal dialysis impact cost and mortality?

**Methods and Data**

The ESRD Population Model was built by an interdisciplinary team at the University of Vermont (UVM) that included experts in clinical care delivery, computational modeling of social systems, public policy, and health economics. The model was also designed in collaboration with the nephrology providers at the University of Vermont Medical Center (UVMMC) in Burlington, Vermont. Through the course of focus groups and the frequent engagement between the social scientists and research clinicians, our team captured the dynamics of the ESRD treatment system and shaped our model output to the interests of the nephrology community. This collaboration led directly to the creation of our model. The stocks and flows making up our model were identified by the nephrology group, and modeled at UVM.

The data for this model currently comes entirely from the US Census Bureau and the USRDS.[11] Calibration for the model involved use of data approximating the year 2020 projection provided by the USRDS in 2009, and several historical scenarios to verify the model produced comparable numbers to historic data for the 2000's.

We employed basic statistic rates provided by the USRDS 2013 Annual Data Report, within the system dynamics structure to create a simple deterministic model. Figure 1 provides a visual representation of the dialysis critical care pathway. Similar diagrams were constructed for transplantation and conservative care pathways as well. The model begins with a stock for the total US population that grows each year at rates projected from Census Bureau data. A fraction of this population is taken as incidence each year and divided between hemodialysis, peritoneal dialysis and the small number of incident transplant patients. The stocks for these three populations interact through dialysis patients receiving transplants, and the common switching of dialysis modes. Finally, mortality rates also act to diminish all three stocks. Financial costs were calculated using annual per patient costs averaged from 2005-2011, collected by the USRDS.[11] Total annual costs are attained as a function of each prevalence stock of patients. Output for the model shows separate breakdowns of costs by patient age, primary diagnosis and treatment type group.

This model captures the complexity of the ESRD field through the interplay of several information loops and the widely varying rates of incidence, transplant and mortality for each of the 35 demographic groups. Appendix A provides a table of the parameters used to calibrate this model. The model is dominated by feed forward loops, rather than the types of feedback loops that generally dominate SD models. The statistical rates of change provided by the USRDS have been used to calibrate our model as proportional changes to stocks, thus the size of the stock at any given moment affects the flow to the next stock. The feedback loop between hemodialysis and peritoneal dialysis, however, is central to the model. Generally, healthier patients tend to use peritoneal dialysis and are better candidates for transplantation. This results in patients on peritoneal dialysis with higher survival rates for their modality. Such trends are reflected in our model. The feedback loop between hemodialysis and peritoneal dialysis reconciles the USRDS data on incidence and prevalence with the provided rates of mortality and transplantation. This system demonstrates the interaction of several component parts of ESRD treatment, covering transplantation, both hemodialysis and peritoneal dialysis, and the transfer of patients between these three major modalities.

**Analysis**

Through focus groups with the UVMMC nephrology team, we identified interventions in three ESRD treatment modalities that could offer opportunities for improving cost efficiency for Medicare and quality of life for patients: transplantation; peritoneal dialysis; and conservative care. These policy intervention simulations were run using our model: a doubling of annual kidney donations; an increase in peritoneal dialysis utilization to 30% from 6.75% of dialysis incidence; and the rerouting of 45% of incident patients 75 and older from renal replacement therapy to conservative care.

**Figure 1.** Example of Dialysis Critical Care Pathway Model

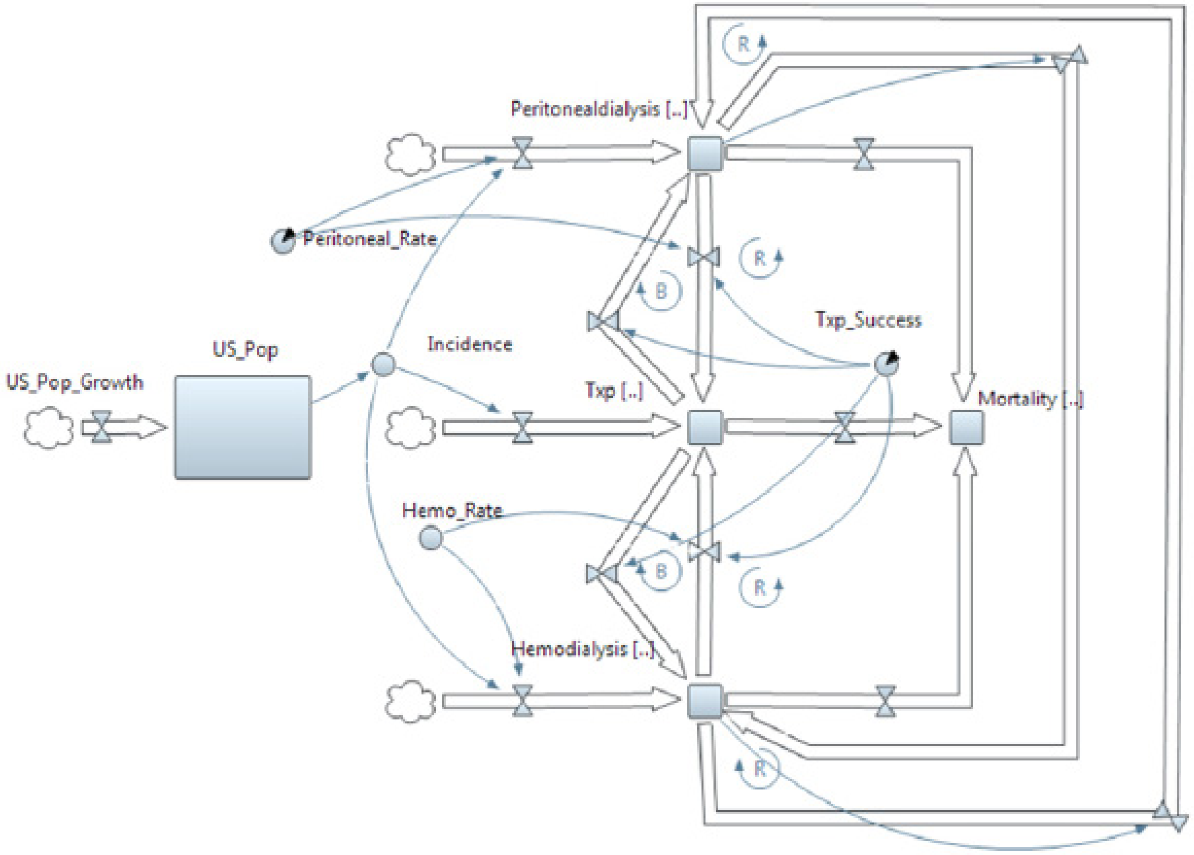

To development the model we noted that rates of transplantation are directly related to rates of organ donation, which are in turn the default opt-in organ donation practice. In other countries where participation is the default and citizens may freely choose to opt-out, donation rates are double those in the United States.[12] As the supply of available kidneys is limited and many patients with ESRD die on the waiting list, we sought to simulate a policy that could double the available supply.

Conservative care—a combination of palliative care and management of anemia and other comorbid conditions—represents a virtually unused option in the United States, even though it may offer a better path for some patients.[13] In 2010, in the United States 21% of dialysis patients—87,541 people—were over age 75. Dialysis patients over age 75 live 2 years on average, compared to younger patients who survive for 3 to 4 years. While a young patient may succumb to kidney failure without dialysis, an elderly patient who may not be eating much could survive as long as 12 to 14 months off of dialysis, depending on their residual renal function.[14] Elderly patients in Australia and Europe who were informed of their situation and given a choice on how to proceed overwhelmingly chose to forego several months of extended life to avoid the low quality of life associated with dialysis treatment.[15,16] The results of these studies have yet to be replicated in the United States, and while attitudes may vary across developed countries, nephrologists in the United States tend to frame treatment as, "'when you will need dialysis,' not if."[17] There are important implications when it is assumed that older patients will start dialysis, including dissatisfaction with care related to poor communication, increased utilization of intensive therapies at the end-of-life due to lack of missed opportunities for addressing quality of life. We believe there is an opportunity here to develop more viable conservative care pathway to more appropriately meet the needs of older adults who do not desire renal replacement therapy. In the simulation we noted conservative care as a critical care pathway.

Finally, home-based dialysis technology is also underutilized. Home-based therapies are less expensive and offer greater flexibility while allowing a patient to live at home and potentially increasing their quality of life. Survival rates for home based therapies are higher than in-center therapy although this may be attributed to the fact that healthier patients select home therapies.[18,19] Home-based strategies are underutilized for two key reasons: 1) the prevalence of private hemodialysis centers and their market power, and 2) home therapies require that the patient performs his/her own care, or have a support system in place (i.e. family, caregiver, etc.). Increased investment in home nursing services and patient and family education and training could nurture an increase in a home dialysis uptake and increased quality of life.

## RESULTS AND CONCLUSIONS

Results of our simulations are compared in scenarios along three metrics: the annual total cost for treating ESRD, the annual average cost per patient, and the annual deaths per thousand patients. Because of the current lack of implementation costs, these metrics will be more or less informative for different policies. For example, because conservative care is currently an undefined pathway in clinical circles, we simply removed patients over age 75 from the pool and their cost and mortality is no longer noted. The impact of conservative care on prevalence and mortality, therefore is exaggerated in our simulation. However, the impact on cost is relatively accurate, as palliative care will incur minimal costs compared to renal replacement therapy.

**Figure 2.** Source USRDS Annual Data Report 2013 and the ESRD Population Model

PD: peritoneal dialysis; CC: conservative care; Txp: transplantation

In 2009, the USRDS expected that if mortality rates continued to decline as they had up until that year, that the actual ESRD prevalence in 2020 would exceed their projection.[6] ESRD mortality has continued to decline, and our projections reflect this trend as well. The most recent published USRDS projection uses data from 2005-2007, while our model uses data from 2007-2011. Taking into account the drop in mortality as well as the slowing in the continued rise of incidence, our model projections for 2020 vary from the USRDS projections by expected margins: 829,185 versus 774,386 for prevalence, 136,623 versus 142,858 for incidence and 109,126 versus 118,617 for mortality.[20,21] Our model also produces detailed output for the incidence, prevalence, mortality, transplantation and financial cost of ESRD by age group, primary diagnosis and treatment type.

The rate of annual deaths per thousand ESRD patients is declining as shown in exhibit one. Adding to the effect caused by the overall increase in prevalence, our three policy interventions, taken individually, or combined, act to improve the odds for ESRD patients for survival, regardless of possible implementation costs. An increase in transplants—the treatment offering the most longevity—predictably offers a larger impact than the next best treatment, increased peritoneal dialysis. The two combined with an increase in conservative care offer almost a one-third reduction in deaths per thousand.

**Figure 3.** Source USRDS Annual Data Report 2013 and the ESRD Population Model

PD:peritoneal dialysis; CC: conservative care; Txp: transplantation

When examining the predicted annual cost of care for ESRD, the conservative care scenario is more impactful because many dialysis patients are removed—and only potentially replaced by vastly less expensive palliative care equivalents—while the benefit of increased survival is fed into the system to mitigate the benefit of decreased costs. The increased use of peritoneal dialysis actually increases total costs long term, despite being a less expensive alternative to hemodialysis because of the increased longevity of peritoneal dialysis patients. This phenomena is only demonstrated over a full 10-year projection, as decreased cost of treatment will initially lower overall costs. However, the increased longevity of patients will eventually overwhelm those savings. These outcomes may be the result of several factors: patients selected for peritoneal dialysis may live longer because they already have more commitment to self-care, and that is a survival advantage. If we assume that doubling the prevalence of peritoneal dialysis will not diminish the quality of patient self-care, increased survival may occur. However, high survival patients are removed from the in–center hemodialysis group, thus diminishing the survivability of that group as a whole. Meanwhile, an increase in transplantation does not result in increased costs—in fact it results in vastly reduced costs—despite an even greater increase in survival, demonstrating the greatly reduced cost of caring for a patient who has received a transplant as compared to a dialysis patient.

Finally, the metric for the annual cost per ESRD patient offers a very different look at conservative care. Peritoneal dialysis resists cost savings again because of increased prevalence from reduced mortality. Transplantation also demonstrates its cost saving potential. Conservative care, however, produces surprising results: minimal cost savings despite a large number of patients being completely removed from the accounting. Possible explanations include the removal of only 45% of what is already a fraction of the total is not enough to effect the system overall, and the possibility that the relativity short longevity of patients over 75 results in lower long-term costs. It is important to stress that conservative care does not mean "no care." These patients are still having outpatient visits, taking medications, and may have nursing home costs. Thus there may be cost shifting: from bundled dialysis costs onto other costs to be considered were not accounted for in the current model.

**Figure 4.** Annual Cost per Patient by Scenario

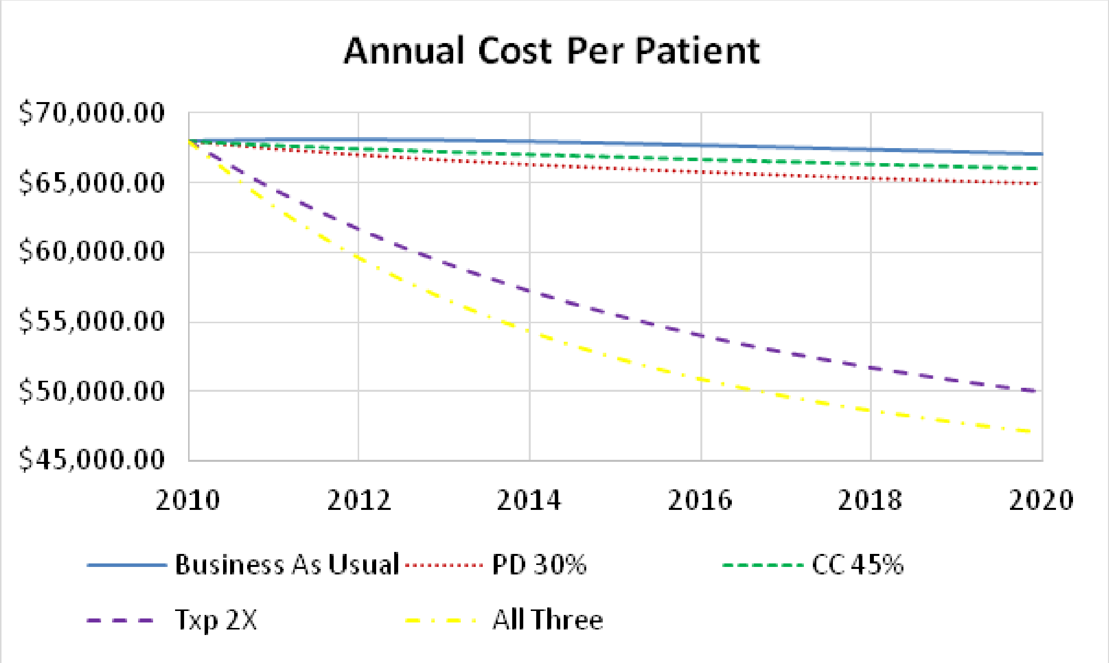

PD: peritoneal dialysis; CC: conservative care; Txp: transplantation

The model suffers from several limitations. The first limitation is the USRDS combining of in-center hemodialysis data with the small number of home hemodialysis patients, as well as the conflation of the two types of peritoneal dialysis in the necessary data sets. The data needed to fit subgroups by age and comorbidity was only available at the level of transplantation, hemodialysis and peritoneal dialysis. Furthermore, true cost-benefit or budget impact analysis are not possible until policy proposals are provided that include the tradeoffs, particularly the financial costs for implementing changes. For example, an increase in the usage of peritoneal dialysis requires more independent candidates for treatment. Inflation is also not accounted for in this model. However, comparing the magnitude of impact on the ESRD population and its growth is possible even without associated costs for change.

## CONCLUSION

Given the ESRD treatment is generally provided under Medicare the potential implications of this model for cost containment under the new Accountable Care Organizations (ACO) framework are worth noting. CMS may look to incentivize ACOs and their clinical care providers to seek alternative clinical care treatment pathways, realizing substantial costs savings and even improving quality care in the process. States looking to contain health care costs may seek to advance "opt out" organ donation policies, provide greater education to older ESRD patients around the conservative care option, and look to improve peritoneal dialysis rates by integrating renal dialysis home care services into "medical home" models of clinical and social service delivery.

We describe this model as an initial proof of concept that will need further refinement with clinical and administrative decisions are to be made based on these projections.

Along with the detailed targeting of our age and primary diagnosis groups, this will make the model ready for use in simulating investment in policy changes for pharmaceuticals companies, hospitals, dialysis centers and state or national health departments. The next stages of model development will be informed by more granular data on patient demographics at the state or regional level.

### Conflict of Interest Declaration

Funding for this project was provided by the University of Vermont Medical Center, Department of Surgery.

### Appendix. Initial and Simulated Values

| | 2005-2011 Average Rate Or 2010 Value | | 2020 Simulated Values | |
|---|---|---|---|---|
| | **N** | **%** | **N** | **%** |
| **Prevalence** | | | | |
| Total* | 596,331 | 100.00 | 829,185 | 100.00 |
| Hemodialysis* | 387,493 | 64.98 | 508,800 | 61.31 |
| Peritoneal Dialysis* | 29,246 | 4.90 | 49,840 | 6.01 |
| Transplant* | 179,592 | 30.12 | 270,546 | 32.50 |
| **Incidence** | | | | |
| Total | 117,390 | 100.00 | 136,623 | 100.00 |
| Hemodialysis | 102,498 | 87.31 | 119,291 | 87.31 |
| Peritoneal Dialysis | 7,557 | 6.44 | 8,796 | 6.44 |
| Transplant | 2,797 | 2.38 | 3,256 | 2.38 |
| **Mortality** | | | | |
| Total | 88,989 | 100.00 | 109,126 | 100.00 |
| Hemodialysis | 78,327 | 88.02 | 94,911 | 86.97 |
| Peritoneal Dialysis | 4,341 | 4.88 | 5,990 | 12.29 |
| Transplant | 6,319 | 7.10 | 8,255 | 5.48 |
| **Transplants** | | | | |
| Dialysis Patients Receiving a Transplant | 12,917 | | 13,937 | |

Source: 2013 USRDS Annual Data Report. *2010 Value; initial model value
